# Editorial: Cerebral localization and neurostimulation for pain

**DOI:** 10.3389/fneur.2022.1019162

**Published:** 2022-09-28

**Authors:** Thomas M. Kinfe, Krishnan V. Chakravarthy, Timothy R. Deer

**Affiliations:** ^1^Division of Functional Neurosurgery and Stereotactic, Department of Neurosurgery, Friedrich-Alexander University (FAU) Erlangen-Nürnberg, Erlangen, Bavaria, Germany; ^2^Department of Anesthesiology, UC San Diego Health Sciences, San Diego, CA, United States; ^3^The Spine & Nerve Centers of the Virginias, Charleston, WV, United States

**Keywords:** spinal modulation, brain pain networks, brain stimulation, pain, connectomic biomarker

Invasive and non-invasive neurostimulation targeting surface and/or deep-seated brain structures, the spinal cord, and peripheral nervous system modulating the somatosensory pain pathway have been applied for the adjunctive treatment of various chronic pain disorders (complex regional pain syndrome, chronic post-stroke pain, facial pain, phantom pain, failed back surgery syndrome, neuropathic pain, trigeminal neuralgia, chronic cancer pain, trigeminal deafferentation pain, anesthesia dolorosa, thalamic pain, spinal cord injury, postherpetic neuralgia, persistent spinal pain syndrome, and plexus brachialis injury) with sustained meaningful responsiveness (1, 2). Despite the fact that evidence-derived data comparing the safety and efficacy of different neurostimulation approaches is limited, neurostimulation targeting nuclei associated with the affective-cognitive (pain attention) brain matrix of pain has gained increased attention as displayed by an increasing number of brain stimulation studies including but not limited to the medial thalamic nuclei (centromedian-parafascicular and central lateral nucleus of the thalamus), anterior cingulate cortex, and anterior limb of the capsule interna/ventral striatum (3). One of the major challenges and open questions in the field is the search for biomarkers (neuroimaging, neurophysiology, molecular, and digital) relevant for target definition most likely to be effective and stimulation parameter adjustment. Neuroimaging and electrophysiological characterizations of the relevant structural and functional properties of the brain pain matrix are lacking and have been mostly assessed in deep brain stimulation for movement disorders, coined as connectome neuromodulation (4, 5). However, the usefulness of these connectome models as a predictive tool for neurostimulation of pain is far from ready for use in a clinical setting, but may serve for the development of personalzed neurostimulation (closed-loop systems; brain sensing technologies) selectively modulating the pain patient pain neural signature. Therefore, a differential view of chronic pain as a multi-network disorder of the brain could potentially impact the diagnosis, patient selection, as well as target and waveform definition of neurostimulation therapies.

Given these gaps, this editorial on the Research Topic *Cerebral Localization and Neurostimulation of Pain* addresses and discusses current and emerging needs in the field of neurostimulation research for pain, presenting and evaluating the most recent advances in neurostimulation for pain research published in 2022. Topics present advances related to the triple network characterization of pain, including the medial and lateral pain pathways along with the descending corticospinal network, neuroimaging of the affective pain matrix defined as the medial thalamus and its projections in pain, the impact of DBS of the anterior cingulate cortex on internal percepts characterized by unexpected auditory hallucinations, and an assay of objective outcome measures for pain using non-invasive spinal tDCS.

De Ridder et al. conceptualized a review and provide an in-depth discussion on the genesis and maintenance of chronic pain. In the past, neuroimaging and electrophysiological studies have mainly contributed to a better characterization of distinct brain areas and networks with rich reciprocal projections. Based on their findings, De Ridder et al. propose a refined definition and framework for chronic pain, distinguishing different networks relevant to sensory, affective-cognitive, and descending pain pathways. This differentiation may be of great relevance for neurostimulation therapies, with the potential to confirm and define novel targets and corresponding stimulation patterns depending on pain phenotyping. Furthermore, this review points to the emerging and unresolved issues of whether a chronic patient is more or less likely to respond to sensory, affective, and/or cognitive neuromodulation and advocates the selective characterization of the brain's pain matrix, which in turn may help to identify suitable neural targets. Brain stimulation techniques that selectively target the affective-cognitive pathways in chronic pain have been applied in a growing number of pain patients, considering the multidimensional characteristics of the brain's pain matrix. This strategy has been supported by neuroimaging studies assessing structural and functional changes in associated networks. For instance, Jin et al. determined activity patterns of the medial thalamus along with an analysis of its projections and correlated their findings with changes in orofacial pain levels. In line with previously published data, decreased activity patterns were detected in the medial thalamus along with a decrease in the projections to the anterior and posterior cingulate cortices, both of which are well known to modulate internal perception and to incorporate sensory and emotional stimuli, hence reinforcing the persistence of pain. These morphological alterations were associated with changes in pain levels, indicating and supporting the putative role of the cingulate cortex in the emotional regulation of perception (pain). Deep brain stimulation of the anterior cingulate cortex, as one of the major projections of the medial thalamic nuclei, was unexpectedly found to evoke music hallucinations in currently running pain trials by Schmittgen et al. Using local field potential measurements in DBS patients, a reduced alpha band and increased gamma band were observed in the anterior cingulate cortex. It is noteworthy that these musical perceptions occurred in the absence of any external auditory stimulus, coined a phantom perception. Similar to chronic pain, these auditory, internally generated perceptions are characterized by common shared pathways compared to chronic pain as a persistent internally generated state. Hypothetically, the prediction error between expectations and perceived external stimuli underpins the therapeutic potential of DBS in the cingulate area to modulate this persistent state of pain. Likewise, invasive spinal cord stimulation has progressed toward novel waveforms such as BurstDR, high-frequency, evoked compound action potential, and differential targeted multiplexed and non-invasive spinal modulation, such as transcutaneous direct current stimulation, and has been increasingly evaluated in observational cohort studies. Interestingly, BurstDR stimulation has been assumed to predominantly interact with medial thalamic circuits, as demonstrated by neuroimaging and electrophysiological studies. Guidetti et al. conceptualized a controlled trial examining the safety and efficacy of tDCS (Th10 anodal/somatosensory cortex cathodal) using objective and subjective outcome measures. Clinically meaningful responsiveness and functional improvements were noted, indicating that tDCS is an effective and safe neurostimulation therapy for chronic pain.

Although the precise mechanism remains unclear, tDCS may modulate aberrant spino-thalamo-cortical circuits despite its impact on the spinal segmental level.

Conclusively, the research data presented in this Special Topic demonstrate the clinical and scientific shift in central neurostimulation for pain toward alternative targets and enhanced waveforms, while also highlighting the long road ahead.

## Author contributions

KC and TD contributing secondary thoughts and editing the final manuscript. All authors conceived and developed the presented ideas and approved the final manuscript.

## Conflict of interest

TK has received training support and works as a consultant for St. Jude Medical, Inc., and works as a consultant for Medtronic Inc. KC is a consultant for Medtronic, Mainstay, Vertos, Rune labs, Nalu medical, Yantra, Higgs Boson Health and Founder at NXTSTIM, Douleur therapeutics, AccuFix Medical. TD is a consultant for Abbott, Saluda, Nalu, Medtronic, SPR, Painteq, Cornorloc, and Spinal Simplicity.

## Publisher's note

All claims expressed in this article are solely those of the authors and do not necessarily represent those of their affiliated organizations, or those of the publisher, the editors and the reviewers. Any product that may be evaluated in this article, or claim that may be made by its manufacturer, is not guaranteed or endorsed by the publisher.
